# Dose–effect relationships in neuroendocrine tumour liver metastases treated with [^166^Ho]-radioembolization

**DOI:** 10.1007/s00259-024-06645-6

**Published:** 2024-02-19

**Authors:** K. Ramdhani, J. Beijer-Verduin, S. C. Ebbers, R. van Rooij, M. L. J. Smits, R. C. G. Bruijnen, H. W. A. M. de Jong, M. G. E. H. Lam, A. J. A. T. Braat

**Affiliations:** Department of Radiology and Nuclear Medicine, University Medical Center Utrecht, Heidelberglaan 100, Huispostnummer E01.132, Utrecht, The Netherlands

**Keywords:** Radioembolization, SIRT, Holmium-166, Neuroendocrine liver metastases, Neuroendocrine tumour

## Abstract

**Purpose:**

Aim of this study was to investigate a dose-response relationship, dose-toxicity relationship, progression free survival (PFS) and overall survival (OS) in neuroendocrine tumour liver metastases (NELM) treated with holmium-166-microspheres radioembolization ([^166^Ho]-radioembolization).

**Materials and methods:**

Single center, retrospective study included patients with NELM that received [^166^Ho]-radioembolization with post-treatment SPECT/CT and CECT or MRI imaging for 3 months follow-up. Post-treatment SPECT/CT was used to calculate tumour (D_t_) and whole liver healthy tissue (D_h_) absorbed dose. Clinical and laboratory toxicity was graded by Common Terminology Criteria for Adverse Events (CTCAE), version 5 at baseline and three-months follow-up. Response was determined according to RECIST 1.1. The tumour and healthy doses was correlated to lesion-based objective response and patient-based toxicity. Kaplan Meier analyses were performed for progression free survival (PFS) and overall survival (OS).

**Results:**

Twenty-seven treatments in 25 patients were included, with a total of 114 tumours. Median follow-up was 14 months (3 – 82 months). Mean D_t_ in non-responders was 68 Gy versus 118 Gy in responders, *p* = 0.01. ROC analysis determined 86 Gy to have the highest sensitivity and specificity, resp. 83% and 81%. Achieving a D_t_ of ≥ 120 Gy provided the highest likelihood of response (90%) for obtaining response. Sixteen patients had grade 1–2 clinical toxicity and only one patient grade 3. No clear healthy liver dose-toxicity relationship was found. The median PFS was 15 months (95% CI [10.2;19.8]) and median OS was not reached.

**Conclusion:**

This study confirms the safety and efficacy of [^166^Ho]-radioembolization in NELM in a real-world setting. A clear dose–response relationship was demonstrated and future studies should aim at a D_t_ of ≥ 120 Gy, being predictive of response. No dose-toxicity relationship could be established.

**Supplementary Information:**

The online version contains supplementary material available at 10.1007/s00259-024-06645-6.

## Introduction

Neuroendocrine neoplasms constitute 2% of all malignancies and comprise a very heterogenous group of tumours with diverse clinical behaviour, histology and responses to treatments [1, 2]. Tumour grading is defined on the WHO/ENETS criteria [3]. Neuroendocrine tumours (NET) with a low or intermediate grade (G1-/G2NET) have a better survival compared to a high-grade NET (G3NET) or neuroendocrine carcinoma. At time of diagnosis, metastases are present in approximately 23% of cases, with the liver being the most common site for distant metastases [4]. As the presence of neuroendocrine liver metastases (NELM) negatively affects survival, safe and effective treatments are necessary to prolong survival. As NELM are supplied by arterial blood supply and commonly present with a multifocal distribution, trans-arterial treatments have gained great interest [5, 6]. Particularly radioembolization, a.k.a. selective internal radiation therapy (SIRT), has shown great promise by reported high tumour objective response rates and limited toxicities. This is further amplified by The European Neuroendocrine Tumour Society (ENETS) guideline from 2016 and the European Society for Medical Oncology (ESMO) guideline from 2020 that have extended the role of radioembolization in NELM, including early application as a tumour debulking treatment or as a salvage treatment in selected cases, after failure of systemic treatments [7, 8]. However, there is a clear absence on dosimetry data in almost all the studies on radioembolization in NELM.

Although the importance of dosimetry in radioembolization has already been clearly demonstrated in the landmark studies in other tumour types (e.g., DOSISPHERE-1 in hepatocellular carcinoma), to date only one study examined dosimetry in NELM [9, 10]. Ebbers et al. established a clear dose-survival and dose–response relationship with a minimum tumour absorbed dose (D_t_) of 150 Gy in NELM patients treated with yttrium-90 [^90^Y] glass microspheres. These studies confirmed that with increasing D_t_, likelihood of objective response will also increase. However, due to the intrinsic differences of commercially available therapeutic particles, dose thresholds of one particle type, cannot be translated one-to-one to another particle [11]. Although holmium-166 [^166^Ho] microspheres and [^90^Y]-microspheres are both used for tumour irradiation, [^166^Ho]-microspheres have better imaging properties. Through emission of gamma photons and being a lanthanide, visualization of distribution of [^166^Ho]-microspheres in the liver and quantification of the absorbed tumour dose on SPECT/CT and MRI is possible. Furthermore, by using a low dose of [^166^Ho]-microspheres in the work-up procedure, the discrepancy between planning and treatment is reduced in comparison to ^99m^Tc-MAA [12].

Therefore, the aim of this study is to examine dose–response relationship and dose-toxicity relationship in NELM treated with [^166^Ho]-microspheres radioembolization ([^166^Ho]-radioembolization).

## Methods

### Patients

All patients from 2012–2022 who underwent [^166^Ho]-radioembolization (Quiremspheres®, Terumo) for NELM were included in this single center, retrospective study. Inclusion criteria were availability of contrast-enhanced computed tomography (CECT) or magnetic resonance imaging (MRI) of the liver at baseline, presence of at least one measurable target lesion according to the Response Evaluation Criteria In Solid Tumours version 1.1 (RECIST 1.1), and presence of a post-treatment [^166^Ho]-SPECT/CT for dosimetric purposes. Need for informed consent was waived by the institutional medical ethics committee for this retrospective cohort study.

### Treatment procedures

All patients had either progressive disease on imaging or deterioration of clinical status due to increased hormone related complaints (e.g., flushing, diarrhoea) prior to ^166^Ho-radioembolization and were discussed in a multidisciplinary tumour board.

In preparation of the radioembolization procedure, 1–3 weeks before treatment an hepatic angiography and administration of [^166^Ho]-scout dose (QuiremScout®, Terumo) or technetium-99 m macroaggregated albumin ([^99m^Tc]-MAA; Pulmocis®, CIS-bio International) was performed in each patient followed by-SPECT/CT. The total amount of administered activity was calculated for the target liver volume, as measured on CECT. Amount of activity prescribed was determined by the treating physicians, which depended on the treatment approach (e.g., whole liver or radiation segmentectomy) and other clinical factors. Administered activity never exceeded the 60 Gy (to the entire liver volume) threshold (i.e., whole liver average absorbed dose), in line with the products instruction for use (IFU). After [^166^Ho]-radioembolization a post-treatment [^166^Ho]-SPECT/CT was acquired 3–5 days later [13]. CECT or MRI at three-months follow-up was performed.

### ^166^Ho SPECT/CT image acquisition

All [^166^Ho]-SPECT/CT’s were acquired and reconstructed on a Symbia T16 (Siemens, Erlangen) that uses a medium-energy low-penetration collimator, on a 128 × 128 matrix (pixel spacing, 4.8 × 4.8 mm), with 120 angles (15 s per projection) over a non-circular 360° orbit. An energy window centred at 81 keV (15% width), together with an additional energy window centred at 118 keV (12% width) to correct the [^166^Ho] photopeak data for down scatter using a window-based scatter correction were used [14]. Low dose non-contrast enhanced CT or CECT images were acquired simultaneously for attenuation correction.

### Dosimetry assessment

Manual image segmentation of the post treatment SPECT/CT was performed in Q-suite version 2.1 (Quirem Medical B.V., The Netherlands), creating volumes of interest (VOIs) on baseline CECT or MRI, or simultaneously acquired CECT during the [^166^Ho]-SPECT/CT. Four distinct compartments were analysed: total liver tissue, target liver tissue (i.e., treatment volume), tumour tissue and total healthy liver tissue (i.e., total liver – tumour). Total counts in the liver in the post-treatment SPECT/CTs were scaled based on the prescribed activity to calculate mean D_t_ of individual tumours, the mean D_t_ of all tumours within the treatment volume of the patient (D_t,all_), the mean absorbed dose in total healthy liver tissue (D_h_). Additionally, pre-treatment total healthy liver volume and percentage (i.e., fraction of total liver), and total tumour volume and percentage were obtained. A minimum tumour diameter of 1 cm was chosen, based on the spatial resolution of [^166^Ho]-SPECT/CT and being measurable disease according to RECIST 1.1. In case the CT scan that was made consecutively with post-treatment SPECT was non-contrast enhanced, [^166^Ho]-SPECT was rigidly registered with baseline CECT or MRI.

### Response assessment

Baseline and three-months follow-up CECT or MRI were assessed according to RECIST 1.1, for both lesions-based and patient-based analysis. A complete response (CR) was achieved if there was a disappearance of the tumour, a partial response (PR) was achieved when there was a decrease in diameter of at least 30%, a progressive response (PD) was characterized by an increase of at least 20%, and stable disease (SD) was a change within 30% decrease or 20% increase of the tumour. Results were also categorized as responders (CR + PR) and as non-responders (SD + PD). In line with official RECIST 1.1 (general RECIST 1.1), patient-based analysis was based on maximum of two hepatic tumours and non-treated and/or extrahepatic disease.

### Toxicity assessment

Relevant clinical and biochemical data at baseline, two weeks, four weeks and at three- months follow-up were retrospectively reviewed according to Common Terminology Criteria for Adverse Events (CTCAE) version 5.0. Laboratory toxicity included albumin, total bilirubin, aspartate aminotransferase (AST), alanine aminotransferase (ALT), gamma-glutamyl transferase (GGT), alkaline phosphatase (APH) and lactate dehydrogenase (LDH). The relative and absolute difference between these laboratory parameters at baseline and after three months of follow-up was also calculated. For clinical toxicity, if symptoms were present at baseline and did not worsen or improve, the toxicity score at follow-up was omitted from the clinical toxicity analysis. If symptoms did worsen, the absolute and highest CTCAE toxicity grade at follow-up was assigned. In cases with a sequential whole liver treatment (first one lobe, several weeks later the other), the highest clinical toxicity grade between sessions or after the second session was used.

### Statistical analysis

Descriptive analyses were used for patient characteristics, therapy specifications, and clinical toxicity. Lesion-based dose–response analysis included a maximum of five tumours per included patient, to avoid the data being skewed by patients with extensive disease.

Patient-based dose–response assessment included the mean dose of all tumours within a patient and general RECIST 1.1. Dose–response relationships were modelled using linear mixed-effects regression models, from which ROC curves were constructed, to incorporate any correlation between tumour doses within a patient. Optimal cut-off point (highest specificity and sensitivity) was calculated with an Youden’s J. Subsequently, a desirable dose was determined on box-plot analysis for the highest specificity (i.e. highest likelihood of response).

On a patient level, radiological PFS was defined as the time elapsed between [^166^Ho]-radioembolization and tumour progression on imaging. OS was assessed as the time between start of the first [^166^Ho]-radioembolization and death due to any cause. Patients were censored if they were still alive at the time of analysis or were lost to follow-up.

Analysis of the relationship between mean D_t,all_ and survival was tested in a Kaplan Meier analysis. Furthermore, dichotomized mean D_t,all_ (cut-off point of 80 and 120 Gy) were tested using log-rank test.

The relationship between CTCAE 5.0 absolute laboratory toxicity grade and total laboratory toxicity score (dependent variables) and total healthy liver percentage and volume and D_h_ (independent variables) were examined using linear regression models. The relationship between these independent variables, absolute, and percentage change in laboratory values were additionally determined by using linear regression models. Additionally, based on previous published data, the cohort was also dichotomized in D_h_ < 30 Gy versus ≥ 30 Gy [15]. Toxicity analyses were not adjusted for confounding. IBM SPSS (v26; IBM, Chicago, USA) and R (v4.3.2; R Core Team 2023) was used to conduct the statistical analysis. P-values < 0.05 were considered to be significant.

## Results

### Study population and treatment characteristics

**Fig. 1:**
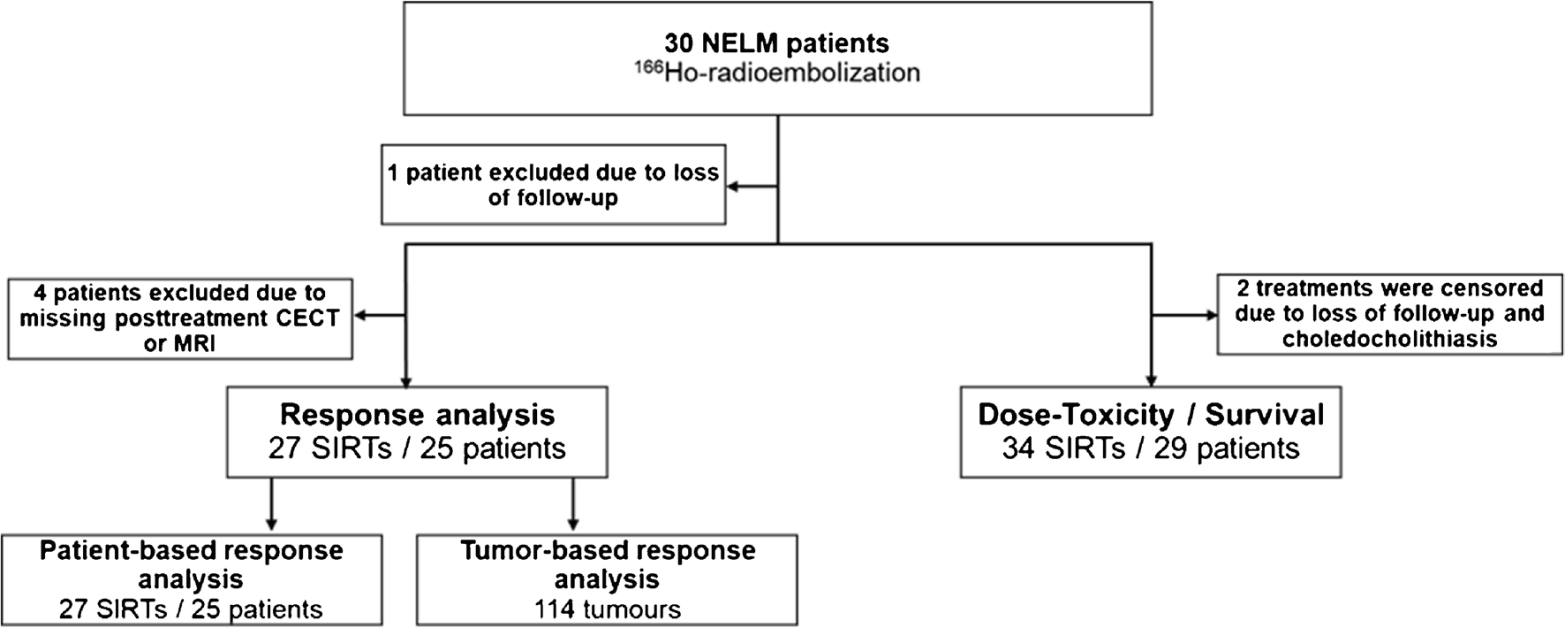
Flowchart of study population

From 2012 – 2022, a total of 30 patients were treated with [^166^Ho]-radioembolization, of whom one patient was excluded for all analyses due to absence of follow-up. All of the remaining 29 patients were included for the dose-toxicity analysis and 25 patients were included for the dose–response analysis, as four patients were excluded due to missing post-treatment CECT or MRI three months after [^166^Ho]-radioembolization (Fig. 1).

One patient had undergone three [^166^Ho]-radioembolization procedures, with an interval of 5 months between the first and second procedure and 4 years between the second and third procedure. Five patients had undergone two [^166^Ho]-radioembolization procedures and 23 patients had undergone one [^166^Ho]-radioembolization procedure (Table 1). A total of 18 treatments (50%) were whole-liver (single session) [^166^Ho]-radioembolization procedures (Table 2).

**Table 1:** Baseline characteristics of all 29 patients included

Mean age (range)	60 (25—80)	Previous treatments	
Male	16 (55.2%)	SSA	23 (79.3%)
Female	13 (44.8%)	PRRT	17 (58.6%)
WHO tumour grade		Primary tumour resection	13 (44.8%)
1	7 (24.2%)	Chemotherapy	7 (24.2%)
2	20 (69.0%)	Hepatic surgery	4 (13.8%)
3	1 (3.4%)	Radiofrequency ablation	2 (6.9%)
Unknown	1 (3.4%)	External beam radiotherapy	2 (6.9%)
Primary tumour origin		Ablation (radiofrequency ablation)	2 (6.9%)
Small bowel	16 (55.2%)	Bland-embolization	1 (3.4%)
Pancreas	7 (24.1%)	Previous [^90^Y]-radioembolization	2 (6.9%)
Lung	4 (13.9%)	Presence of ascites	1 (3.4%)
Gastric	1 (3.4%)	Presence of extrahepatic metastases	18 (62.1%)
Unknown primary	1 (3.4%)	Number of [^166^Ho]-radioembolization treatments	
Functional tumour	17 (58.6%)	1	23 (79.3%)
Child–Pugh score		2	5 (17.3%)
A	27 (93.1%)	3	1 (3.4%)
B	2 (6.9%)	Median follow-up duration in months (range)	14 (3—82)

*WHO*  world health organisation, *SSA* somatostatin analogues, *PRRT* peptide receptor radionuclide therapy

**Table 2:** ^166^Ho-radioembolization specifics (*n* = 36)

Total administered activity (MBq)—median (range)	4.957 (1.256- 11.089)
Treatment distribution	
Whole liver (single session)	18 (50.0%)
Sequential whole liver	3 (8.3%)
Unilobar	12 (33.4%)
Segmental	3 (8.3%)
Hepatic tumour burden in %—median (range)	14.0 (0.2–69.6)
Hepatic tumour burden in cm^3^—median (range)	272 (3–2470)
Healthy liver in %—median (range)	86.0 (30.4—99.8)
Healthy liver volume in cm^3^—median (range)	1654 (841—2832)
Median absorbed dose in healthy liver tissue* (D_h_) in Gy (range)	32 (9—48)
Median absorbed tumour dose* (D_t_) in Gy (range)	71 (37–172)

*patient-based analysis

Of the included patients, 55% had a small-intestinal NET, 69% a WHO grade 2 NET, previously treated with somatostatin analogues (SSA; 79%) and/or peptide receptor radionuclide therapy (PRRT; 59%). None of the patients were treatment naïve (Table 1).

### Dose–response assessment

Lesion-based dose–response analysis included 25 patients with 27 treatments, with a total of 114 tumours. The median tumour size was 2.7 cm (range 1.0 – 12.7 cm).

**Fig. 2:**
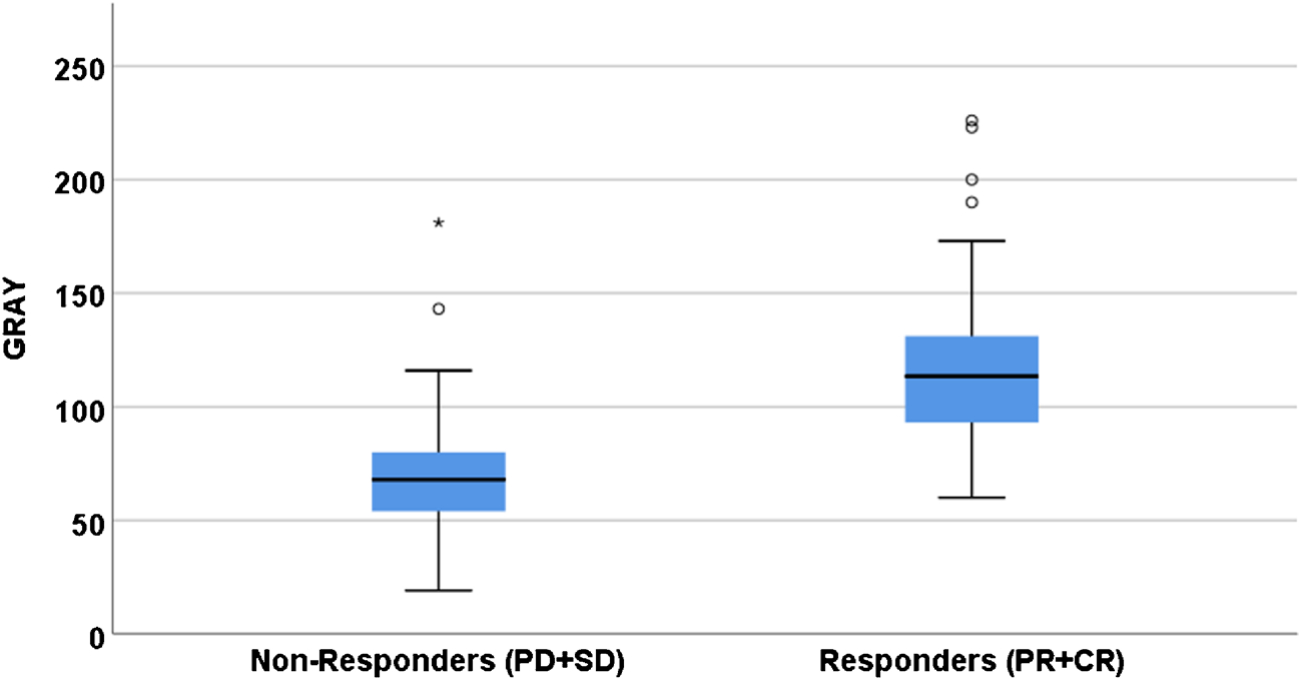
Per lesion analysis of dose–response relationship. Boxplot showing the relationship between tumour absorbed dose and response on tumour level. PD/SD; progressive disease/stable disease, PR/CR; partial response/complete response. *p* = 0.01

**Fig. 3:**
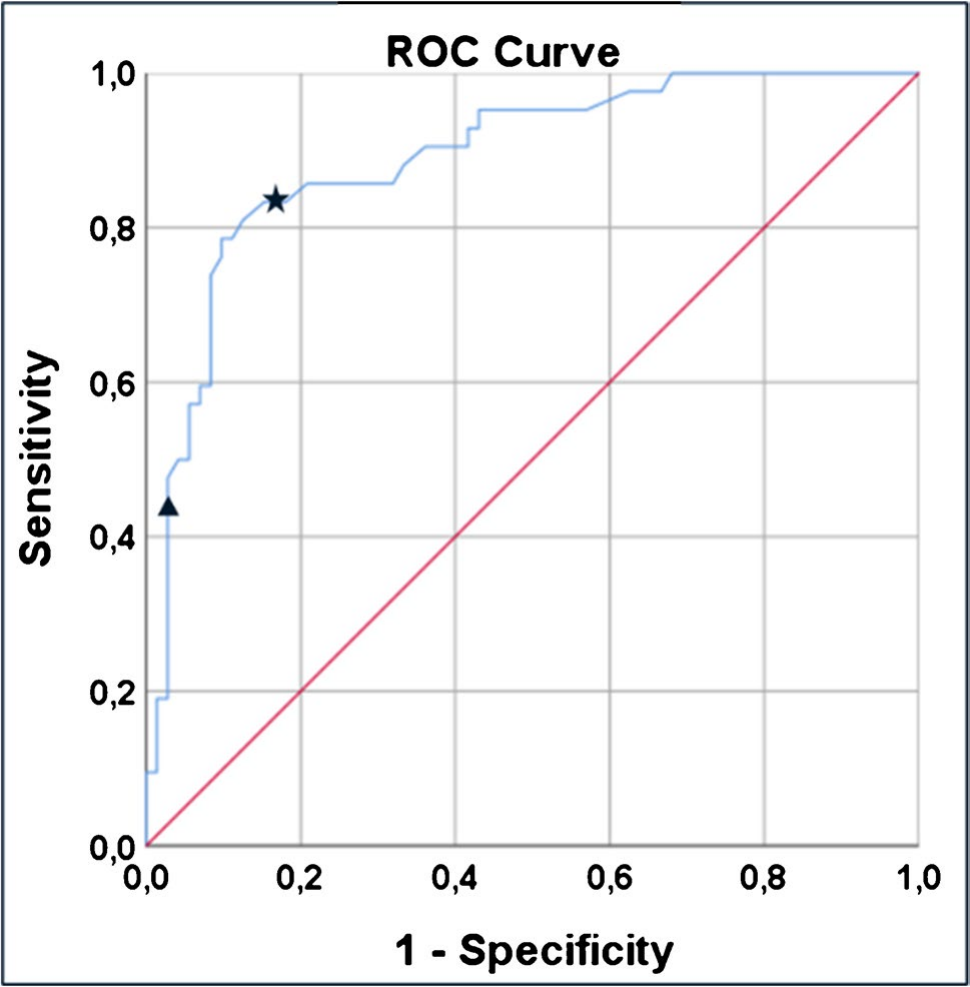
Receiver operating characteristic curve depicting the accuracy of the log-transformed dose in predicting response. With the black star marking Youden-J and the black triangle marking 120 Gy with the highest specificity

Six tumours had CR, 36 tumours had PR, 69 tumours had SD and three tumours had PD, resulting in an objective response rate (ORR) of 37% and disease control rate (DCR) of 97% of target lesions. The mean D_t_ in non-responding tumours (PD + SD) was 68 Gy versus 118 Gy in responding tumours (CR + PR) (*p* = 0.01; Fig. 2). In the ROC analysis, Youden’s-J was 86 Gy to have the highest sensitivity and specificity, resp. 83% and 81%.%. The area under the curve (AUC) was 0.891 (95% CI [0.83; 0.95]). A D_t_ of 120 Gy had the highest specificity (97%) and provided the highest likelihood of response (90%; Fig. 3).

**Fig. 4:**
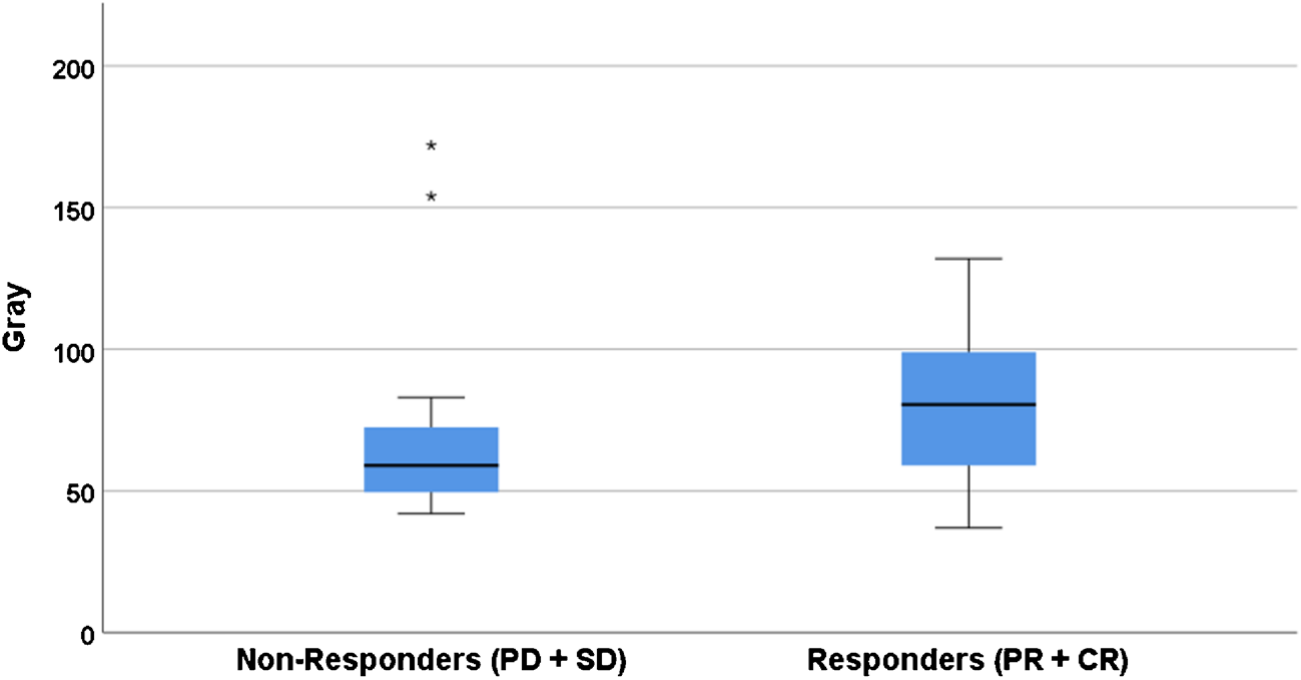
Dose–response relationship on patient level. Boxplot showing the relationship between tumour absorbed dose and response on patient level. PD + SD; progressive disease + stable disease; PR + CR; partial response + complete response. *p* = 0.7

In the patient-based analysis, 14 patients had PR, 2 patients had SD and 9 patients had PD, resulting in an ORR of 56% and DCR of 64%. Dose–response analysis on patient level did not show a significant difference, with a mean D_t,all_ of 76 Gy in non-responders (*n* = 11) versus 81 Gy in responders (*n* = 14; *p* = 0.7; Fig. 4).

### Dose-toxicity assessment

A total of 29 patients, who underwent a total of 36 treatments, were analysed for the dose-toxicity assessment. Two of the 36 treatments were excluded due to loss of follow-up and non-[^166^Ho]-radioembolization related choledocholithiasis.

A total of 14 patients (16 treatments) had clinical toxicity and 15 patients (18 treatments) reported no clinical toxicity. Only one grade 3 toxicity was reported, the remaining toxicities included five grade 2 toxicities and 10 grade 1 toxicities. No new grade 4–5 toxicity or carcinoid crisis was encountered. No dose-toxicity relationship was found (p > 0.05).

Laboratory toxicity was reported in 20 patients (24 treatments). Four grade 3, four grade 2 and 16 grade 1 laboratory toxicity was reported (Table 3). No dose-toxicity relationship was found (p > 0.05).

**Table 3:** Newly identified toxicity in patients within 3 months after treatment, compared to baseline. *REILD; radioembolization-induced liver disease*

	CTCAE					
Biochemical toxicity	**0**	**1**	**2**	**3**	**4**	**5**
APH	24	8	1	1	0	0
ALAT	24	8	2	0	0	0
ASAT	20	13	1	0	0	0
Albumin	30	2	2	0	0	0
GGT	19	8	5	2	0	0
LDH	30	4	0	0	0	0
Bilirubin	31	3	0	0	0	0
Healthy liver mean dose (Dh)	**0**	**1**	**2**	**3**	**4**	**5**
< 30 Gy	6	4	3	1	0	0
> 30 Gy	4	12	1	3	0	0
Clinical toxicity	**0**	**1**	**2**	**3**	**4**	**5**
Fatigue	27	3	4	0	0	0
Abdominal pain	28	5	1	0	0	0
Nausea	30	1	3	0	0	0
Vomiting	33	1	0	0	0	0
Pain, other	32	2	0	0	0	0
Cramping hands	33	1	0	0	0	0
Dizziness	32	1	1	0	0	0
Dyspnea	33	1	0	0	0	0
Liver abcess	33	0	0	1	0	0
Flushing	0	3	0	0	0	0
REILD	0	0	0	0	0	0
Healthy liver mean dose (Dh)	**0**	**1**	**2**	**3**	**4**	**5**
< 30 Gy	12	1	1	0	0	0
> 30 Gy	6	10	3	1	0	0

Clinical and laboratory toxicity and healthy liver mean dose (D_h_) dichotomized in < 30 Gy and > 30 Gy

Of the treatments with D_h_ < 30 Gy one patient (7%) experienced grade 2 clinical toxicity ( nausea, flushing and fatigue; Table 3) and laboratory toxicity was experienced in seven patients (eight treatments, 57% Table 3) with one grade 3 toxicity (increased APH), three grade 2 (GGT and Albumin) and four grade 1 toxicities (AST, GGT, APH and bilirubine).

Of the treatments with D_h_ ≥ 30 Gy, 70% experienced clinical toxicities (Table 3), with one grade 3 toxicity (liver abscess, which was treated with intravenous antibiotics) and 80% experienced laboratory toxicities (Table 3), of which three grade 3 (increased AST, GGT and APH).

In the linear regression models, no dose-toxicity relationship was found. However, after dichotomizing (D_h_ < 30 Gy versus ≥ 30 Gy), a significant dose-toxicity relationship in clinical toxicity (*p* = 0.016; supplemental Table 1) was found. None of the analyses showed a dose-toxicity relationship for laboratory toxicity, even after dichotomization (*p* = 0.501; supplemental Table 1).

### Survival Analysis

**Fig. 5:**
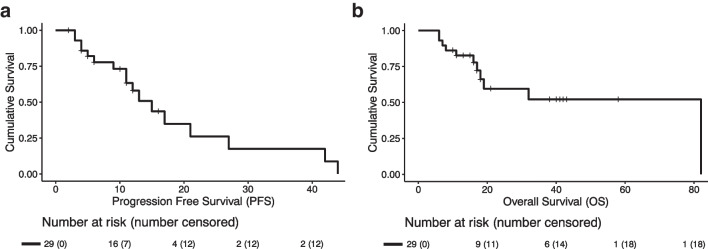
**a** Survival curve for PFS in which 12 patients were censored. Median overall PFS was 15 months (95% CI [10.2;19.8]). There was no significant association between Mean D_t,all_ and PFS. **b** Survival curve for OS. Mean OS was 50.5 months (95%CI [34.7;66.3]), 18 patients were censored and median OS was not reached. There was no significant association between mean D_t,all_ and OS

Median follow-up time was 14 months (3–82 months) for the 29 patients included. Median overall PFS was 15 months (95% CI [10.2;19.8]). Twelve patients (41%) were censored (seven not reached and five lost-to-follow-up) (Fig. 5).

Mean D_t,all_ was not significantly associated with progression free survival when dichotomizing the patients in < 80 Gy versus ≥ 80 Gy (13 vs. 17 months; *p* = 0.9) or < 120 Gy versus ≥ 120 Gy (13 vs. 42 months; p > 0.5; supplemental Fig. 1).

Median OS was not reached (Fig. 5). In the OS analyses, dichotomization in < 80 Gy versus ≥ 80 Gy or < 120 Gy versus ≥ 120 Gy, yielded no significant association (p > 0.5; supplemental Fig. 2).

## Discussion

This study is the first to investigate a dose–response relationship, and a dose-toxicity relationship in NELM treated with [^166^Ho]-radioembolization. A clear dose–response relationship was demonstrated with a D_t_ of ≥ 120 Gy showing a high 90% positive predictive value for an objective response according to RECIST 1.1.

There was no significant difference in D_t,all_ between responders and non-responders in the patient-based analysis and no linear dose-toxicity relationship was found. However, patients with a D_h_ < 30 Gy showed significantly less short-term clinical toxicity (*p* = 0.016).

In this cohort, median PFS was 15 months (95% CI [10.2;19.8]) and median OS was not reached. PFS and OS were found not to be significantly associated with mean D_t,all_, however number of patients in this analysis was limited.

Earlier studies demonstrated dose thresholds in patients with other tumour types treated with [^166^Ho]-radioembolization. Bastiaannet et al. conducted a prospective study in 36 patients with liver metastases of various origins (with only one patient having NELM). They demonstrated a significant difference in tumour-absorbed dose between CR and SD (232 Gy vs 147 Gy; *p* = 0.01) and between CR and PD (232 Gy vs 117 Gy; *p* = 0.0008) [16]. A follow-up study by Roekel et al. in 40 patients with colorectal liver metastases (mCRC) also demonstrated a significant dose–response relationship. The authors advocated to personalize the administered treatment dose to achieve a mean D_t,all_ of > 90 Gy [17]. The present study showed a dose threshold in [^166^Ho]-radioembolization in NELM, as previously shown by Ebbers et al. for [^90^Y]-glass radioembolization. In the present cohort study with [^166^Ho]-radioembolization, a D_t_ of ≥ 120 Gy resulted in 90% likelihood of objective response. For [^90^Y]-glass radioembolization, the authors proposed a D_t_ of > 150 Gy. The difference in dose thresholds can likely be explained by intrinsic differences of the products used, especially differences in specific activity of the used microspheres and differences in physical half-life. This was recently emphasized by the EANM guideline that specifically stated (the need for) different dose thresholds for the different microspheres and for different tumour types [11].

Ebbers et al. suggested a patient-based dose survival relationship using a dose threshold of 150 Gy using [^90^Y]-glass microspheres, however this was not sustained after correction for confounders [10]. In this study, a patient-based dose survival relationship could not be established. An explanation for lack of significance in the patient based dose–response analysis is due to the multitude of tumours within a patient and the spatial resolution of [^166^Ho]-SPECT/CT, in which D_t_ in small tumours(< 1 cm) are not accounted for.

There was no dose-toxicity relationship in our study. Van Roekel et al. advocated a dose threshold with a D_h_ of < 55 Gy in mCRC. All the patients in the current study had a calculated D_h_ below this threshold (all < 50 Gy), and no radioembolization- induced liver disease (REILD) was encountered. The suggested threshold by van Roekel et al. cannot be directly translated to NELM due to intrinsic tumour differences, differences in disease course, differences in treatment algorithm etc. A recent study by Stella et al. investigated automatic healthy liver segmentation in 31 patients with NELM who were treated with [^166^Ho]-radioembolization sequentially after PRRT (i.e., HEPAR PLuS study). They also found no dose-toxicity relationship [15, 18]. In that post-hoc analysis study, all patients had D_h_ of ≤ 35 Gy and only two cases of significant hepatoxicity were reported, respectively one REILD case with a D_h_ of 30 Gy and one patient with a CTCAE grade 4 toxicity, with a D_h_ of 23 Gy. The authors concluded that no conclusions can be drawn on the limited occurrence of hepatotoxicity. Although our study did not find a significant dose-toxicity relationship, significantly less (clinical) toxicity was reported when D_h_ was < 30 Gy. In line with the presence of dose–response relationships, these results indirectly suggest the presence of a dose-toxicity relationship. Disease course of patients suffering from NELM is significantly different compared to other tumour types (e.g., mCRC), thus one may adhere to this suggested 30 Gy D_h_ threshold to remain conservatively safe. However, the current study showed no major toxicities with a D_h_ up to 50 Gy. The current study is underpowered due to lack of significant toxicity.

Correlation to mean dose to the whole non-tumour liver tissue (D_h_) was deliberately chosen, as in previous studies on HCC (also hypervascular disease), D_h_ is more predictive for post-treatment toxicity than mean dose to the non-tumour target volume alone [19, 20]. On the one hand, in first-line setting less toxicity is accepted as other subsequential treatments (e.g. PRRT) are still readily available. On the other hand, patients in salvage setting, having a worse prognosis, more toxicity may be accepted. Nonetheless, future studies on ^166^Ho-microsphere radioembolization should provide more insights into acceptable D_h_ threshold.

This cohort study further confirmed the results published on radioembolization in patients with NELM, as reported median PFS and OS in other studies vary between 16 – 25 months and 29 – 35 months, respectively [5]. The median PFS of 15 months in this cohort study fits within that range. Median OS in the current study was not reached. In our survival analysis, mean D_t,all_ was not significantly associated with PFS or OS. However, a trend can be noted in the analyses dichotomizing for a mean D_t_ ≥ 120 Gy, giving the highest PFS and OS. This may resemble the previously published study by Ebbers et al. who showed an improved survival in NELM patients treated with [^90^Y]-glass microspheres, receiving D_t,all_ ≥ 150 Gy compared to < 150 Gy, 29 versus 12 months (*p* = 0.018), respectively. However, the current study was underpowered to draw a firm conclusion.

The patient-based ORR at three months of 37% in this cohort study is in line with previous studies (16%-50%) and with the previously published HEPAR PLuS study (43%) [5, 21]. In the NETTER-1 trial on [^177^Lu]Lu-DOTATATE with long-acting octreotide, ORR at three months was only 18%.The current cohort study demonstrates the effectiveness of [^166^Ho]-radioembolization as either a debulking or salvage treatment, confirming the current place of radioembolization in both the ENETS and ESMO guidelines [7, 8]. The application of D_t_ > 120 Gy threshold with prospective dosimetry in future studies will probably show further improvement of outcomes.

This study had several limitations. Besides being a retrospective study and relatively small population, the chosen follow-up period for radiological response assessment of three months was relatively short. Especially considering that studies have shown that up to 25% of response assessments can change at six months [22]. In line with other studies on radioembolization, the included patients were heterogeneous (different tumour grade, different tumour origins, etc.), had different lines of previous treatments and none were treatment naïve, therefore creating bias. Follow-up duration in this cohort study was relatively short, limiting the number of events in our survival analyses (in particular OS). Due to the limited number of patients, the survival analyses were not corrected for known clinical factors (e.g. tumour grading, etc.). Number of reported and encountered adverse events was limited, illustrating that radioembolization is a safe and effective treatment, but probably also suffers from a reporting bias in a retrospective setting. Long-term toxicity, and especially long term hepatotoxicity was not investigated in this current study. Concerns have been raised in the past, mainly in North America, after cirrhosis-like morphology (without clinical complaints) was reported on imaging studies in patients treated with radioembolization.

However in a prospective study and larger retrospective studies these concerns could not be affirmed. Furthermore, a recent single center study, demonstrated no significant difference (22% vs. 29%, respectively) in long-term hepatotoxicity between patients treated with TACE and radioembolization [5].

This is the first study to demonstrate a dose–response relationship with [^166^Ho]-radioembolization. Prospective studies are necessary to further evaluate [^166^Ho]-radioembolization in treatment of NELM. These must incorporate prospective dosimetry and individualized treatment planning. Besides dosimetry, future studies should include longer follow-up than in radioembolization studies in other tumour types.

As inclusion of prospective dosimetry in multicenter studies is challenging, the suggested [^166^Ho]–[^99m^Tc] dual-isotope SPECT/CT by Stella et al. may ease translation and broad implementation [15]. They concluded that automatic segmentation of the healthy liver tissue could optimize [^166^Ho]-radioembolization, as the total healthy liver dose is dose-limiting, thus accurate assessment of D_h_ is essential to mitigate possible hepatotoxicity in NELM.

## Conclusion

This study confirms the safety and efficacy of [^166^Ho]-radioembolization in NELM in a real-world setting, with a clear dose–response relationship. Future prospective studies should include prospective dosimetry and aim for a tumour absorbed dose of at least 120 Gy. No dose-toxicity relationship could be demonstrated.

### Supplementary Information

Below is the link to the electronic supplementary material.

Supplementary file1 (PDF 120 KB)

## Data Availability

The data that support the findings of this study are available upon reasonably request via contacting the corresponding author.
