# Supplementary material for: Dose–effect relationships in neuroendocrine tumour liver metastases treated with [166Ho]-radioembolization
Source: Eur J Nucl Med Mol Imaging. 2024 Feb 19;51(7):2114–23. doi: 10.1007/s00259-024-06645-6 (PMC11139696; doi:10.1007/s00259-024-06645-6)
Supplement: Supplementary file 1 — Supplementary file1 (PDF 120 KB) [file 259_2024_6645_MOESM1_ESM.pdf]

Supplemental.

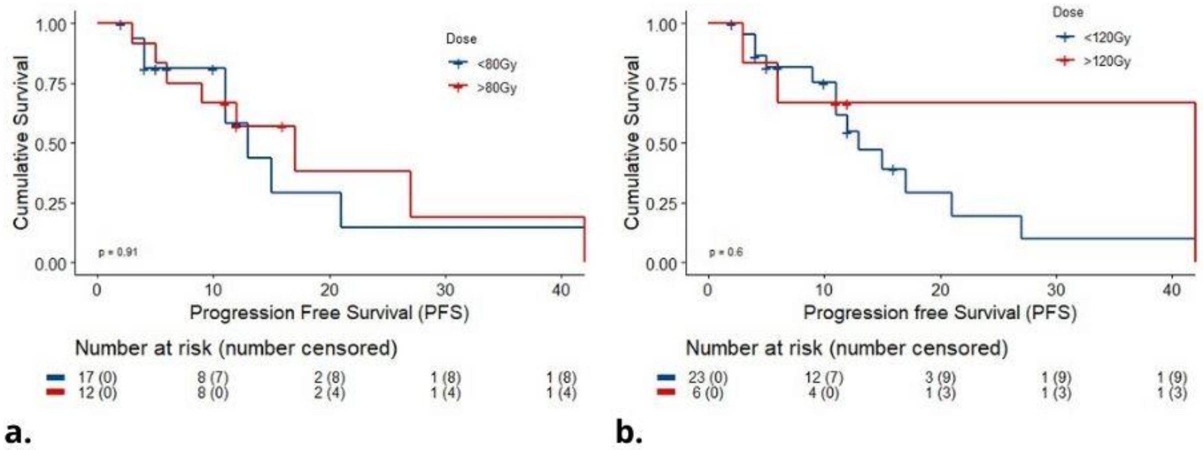

Fig. 1 Supplemental: Survival curves for mean tumour absorbed dose and disease progression, per-patient analysis. **a.** Difference in PFS between patients receiving an overall average tumour absorbed dose above or below 80 Gy. **b.** Difference in PFS between patients receiving an overall average tumour absorbed dose above or below 120 Gy.

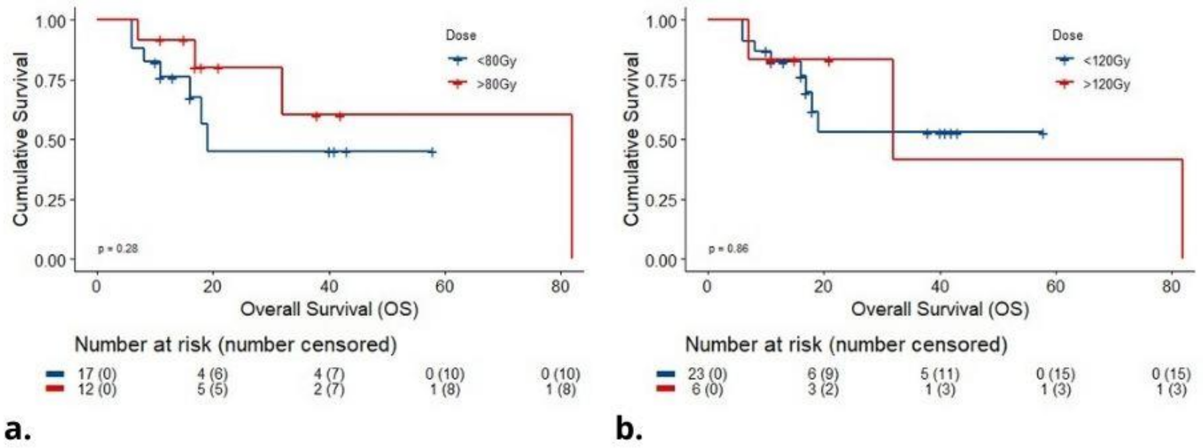

Fig. 2 Supplemental: Survival curves for mean tumour dose and overall survival, per-patient analysis. **a.** Difference in OS between patients receiving an overall average tumour absorbed dose above or below 80 Gy. **b.** Difference in OS between patients receiving an overall average tumour absorbed dose above or below 120 Gy.

Clinical Toxicity

	B	S.E.	Wald	df	Sig.	Exp(B)
CTCAE	1,673	,693	5,820	1	,016	5,328

Labarotory Toxicity

	B	S.E.	Wald	df	Sig.	Exp(B)
CTCAE	,259	,385	,453	1	,501	1,296

Table 1 Supplemental: Logistic regression model with dichotomizing healthy liver mean dose (Dh) in in <30 and > 30 Gy for clinical and laboratory toxicity.
